# THE IMPLICATIONS OF DIGITAL SOCIAL INTERACTIONS FOR OLDER ADULTS’ EXPERIENCES OF WELL-BEING IN DAILY LIFE

**DOI:** 10.1093/geroni/igz038.053

**Published:** 2019-11-08

**Authors:** Shannon T Mejia, Shelbie Turner, Karen Hooker

**Affiliations:** 1 University of Illinois, Urbana-Champaign, Champaign, Illinois, United States; 2 Oregon State University, Corvallis, Oregon, United States

## Abstract

Digital communication technologies expand opportunities for social interactions and as a result have the potential to either amplify or dampen the coupling of social interactions with well-being in daily life. We use data from the 100-day Personal Understanding of Life and Social Experiences project (n = 99, age = 50 – 88) to examine variation in the sensitivity of older adults’ daily reports of well-being to the quality of social interactions with their five closest social partners across digital (email/social media) and analogue (in person/by phone) interactions. Digital interactions were more common among less-close social partners. Multilevel random coefficient models showed days with more digital interactions than normal to be characterized by a) lower well-being and b) less sensitivity in well-being to the quality of social interactions with close social partners on that day. The implications of our findings are discussed within a life-span perspective of social relationships and well-being.

